# Purine DNA Lesions at Different Oxygen Concentration in DNA Repair-Impaired Human Cells (EUE-siXPA)

**DOI:** 10.3390/cells8111377

**Published:** 2019-11-01

**Authors:** Marios G. Krokidis, Eleonora Parlanti, Mariarosaria D’Errico, Barbara Pascucci, Anna Pino, Alessandro Alimonti, Donatella Pietraforte, Annalisa Masi, Carla Ferreri, Chryssostomos Chatgilialoglu

**Affiliations:** 1 Istituto per la Sintesi Organica e la Fotoreattività, Consiglio Nazionale delle Ricerche, Via P. Gobetti 101, 40129 Bologna, Italy; m.krokidis@inn.demokritos.gr (M.G.K.); annalisa.masi@isof.cnr.it (A.M.); carla.ferreri@isof.cnr.it (C.F.); 2 Institute of Nanoscience and Nanotechnology, N.C.S.R. “Demokritos”, 15310 Agia Paraskevi Attikis, Athens, Greece; 3 Department of Environment and Health, Istituto Superiore di Sanità, Viale Regina Elena 299, 00161 Rome, Italy; eleonora.parlanti@iss.it (E.P.); mariarosaria.derrico@iss.it (M.D.); barbara.pascucci@ic.cnr.it (B.P.); anna.pino@iss.it (A.P.); alessandro.alimonti@iss.it (A.A.); 4 Institute of Crystallography, Consiglio Nazionale delle Ricerche, Monterotondo Stazione, 00185 Rome, Italy; 5 Core Facilities, Istituto Superiore di Sanità, Viale Regina Elena 299, 00161 Rome, Italy; donatella.pietraforte@iss.it; 6 Center for Advanced Technologies, Adam Mickiewicz University, 61-614 Poznań, Poland

**Keywords:** XPA, DNA repair, hypoxia, oxidative lesions, hydroxyl radicals, oxygen concentration

## Abstract

Xeroderma Pigmentosum (XP) is a DNA repair disease characterized by nucleotide excision repair (NER) malfunction, leading to photosensitivity and increased incidence of skin malignancies. The role of XP-A in NER pathways has been well studied while discrepancies associated with ROS levels and the role of radical species between normal and deficient XPA cell lines have been observed. Using liquid chromatography tandem mass spectrometry we have determined the four 5’,8-cyclopurines (cPu) lesions (i.e., 5′*R*-cdG, 5′*S*-cdG, 5′*R*-cdA and 5′*S*-cdA), 8-oxo-dA and 8-oxo-dG in wt (EUE-pBD650) and XPA-deficient (EUE-siXPA) human embryonic epithelial cell lines, under different oxygen tension (hyperoxic 21%, physioxic 5% and hypoxic 1%). The levels of Fe and Cu were also measured. The main findings of our study were: (i) the total amount of cPu (1.82–2.52 lesions/10^6^ nucleotides) is the same order of magnitude as 8-oxo-Pu (3.10–4.11 lesions/10^6^ nucleotides) in both cell types, (ii) the four cPu levels are similar in hyperoxic and physioxic conditions for both wt and deficient cell lines, whereas 8-oxo-Pu increases in all cases, (iii) both wt and deficient cell lines accumulated high levels of cPu under hypoxic compared to physioxic conditions, whereas the 8-oxo-Pu levels show an opposite trend, (iv) the diastereoisomeric ratios 5′*R*/5′*S* are independent of oxygen concentration being 0.29 for cdG and 2.69 for cdA for EUE-pBD650 (wt) and 0.32 for cdG and 2.94 for cdA for EUE-siXPA (deficient), (v) in deficient cell lines Fe levels were significantly higher. The data show for the first time the connection of oxygen concentration in cells with different DNA repair ability and the levels of different DNA lesions highlighting the significance of cPu. Membrane lipidomic data at 21% O_2_ indicated differences in the fatty acid contents between wild type and deficient cells, envisaging functional effects on membranes associated with the different repair capabilities, to be further investigated.

## 1. Introduction

**Figure 1:**
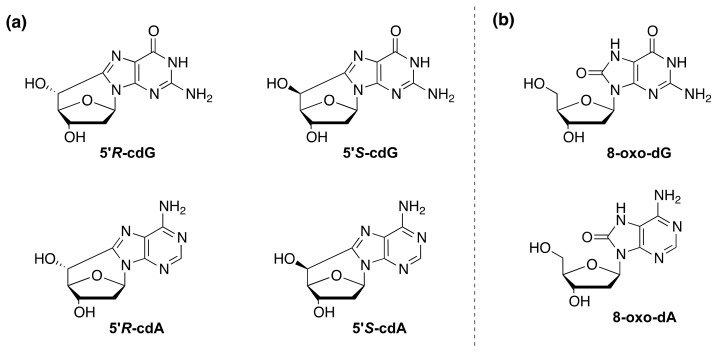
(**a**) Structures of 5′,8-cyclo-2′-deoxyguanosine (cdG) and 5′,8-cyclo-2′-deoxyadenosine (cdA) in their 5’*R* and 5’*S* diastereomeric forms. (**b**) Structure of 8-oxo-2′-deoxyguanosine (8-oxo-dG) and 8-oxo-2′-deoxyadenosine (8-oxo-dA).

Xeroderma Pigmentosum (XP-complementation group A–G) is a rare autosomal recessive disorder, which presents itself with an increased incidence of sun-induced skin cancer and of internal cancer [1]. Patients of the complementation group XP-A also are prone to neurodegeneration. Neurological defects appear between 2 and 8 years of age, with microcephaly, mild cognitive impairment followed by cerebellar alterations and, later, neuropathy [2]. XP-A is one of the 30 proteins involved in the nucleotide excision repair (NER), a repair pathway responsible for the removal of those lesions that distort the DNA helix. NER operates by two distinct pathways: global genome repair (GGR) that removes lesions from the genome overall and transcription-coupled repair (TCR) that repairs transcriptionally active domains [3]. XP-A plays a central role in both TCR and GGR. In differentiated cells, XP-A is involved in the transcription-domain associated repair (DAR), a DNA repair system that repairs bulky lesions in both transcribed and non-transcribed strands of active genes, whereas NER is quite absent in non-transcribed genes [4]. It is increasingly evident that NER factors are also involved in the repair of oxidatively generated DNA damage [5]. These lesions are prevalently repaired by the base excision repair (BER), involving specific DNA glycosylases in the removal of damaged bases [6]. In a previous study some of us showed that the XPC-HR23B complex acts as a BER cofactor for 8-oxo-7,8-dihydro-2′-deoxyguanosine (8–oxo-dG, Figure 1b) by stimulating the activity of its specific DNA glycosylase OGG1 [7]. Recently, some of us demonstrated that primary fibroblasts derived from XPA patients accumulate 8–oxo-dG in their genome [8], as already shown in primary fibroblasts derived from XPC patients and from Cockayne Syndrome patients (A and B complementation groups) [7,9]. These findings suggested a functional overlap between XPA and XPC in the removal of 8–oxo-dG [8].

Another family of thoroughly investigated oxidative lesions, also for their role in neurodegenerative disease are the purine 5’,8-cyclo-2’-deoxynucleosides (cPu). The 5′,8-cyclo-2′-deoxyadenosine (cdA) and 5′,8-cyclo-2′-deoxyguanosine (cdG) exist in 5’*R* and 5’*S* diastereoisomeric forms (Figure 1a). The peculiarity of these lesions is that they are only repaired by the NER pathway [10,11,12,13] with increased efficiency for both 5′*R* diastereoisomers compared to 5′*S*, by a factor of 2 [14,15,16], whereas they are not removed by BER [17,18]. In contrast with other oxidative DNA lesions, cPu are exclusively generated by hydrogen atom abstraction from the 2-deoxyribose units by HO^•^ radical. These lesions were found to be: chemically stable [19,20], endogenously formed in mammalian cellular DNA [21,22,23,24,25], repaired exclusively by the NER pathway [14,18,26], strongly, but not totally, cause of blockage of the transcription by RNA polymerase II which occurs in cells derived from XP patients [17,27].

**Figure 2:**
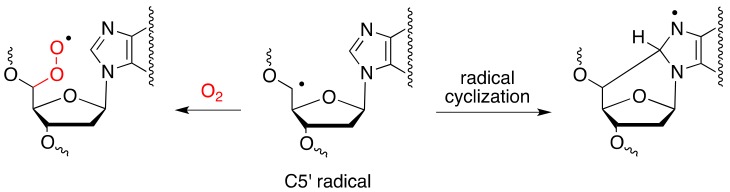
Partition of C5′ radical between cyclization and addition to molecular oxygen.

It is well known that the concentration of O_2_ can strongly influence the formation of cPu [28,29,30,31]. Gamma-radiolysis experiments under modulated aerobic conditions were carried out to investigate the formation of cPu in calf thymus DNA [30,31]. It was found that the C5′ carbon radical is converted quantitatively to cdA and cdG in the absence of oxygen, whereas the formation of cPu decreases in the presence of oxygen, indicating competition paths for C5′ radical between cyclization and addition to oxygen (Figure 2).

The main processes that generate endogenously HO^•^ radicals are depicted in reactions (1)–(3): the Fenton reaction of H_2_O_2_, the reduction of HOCl by the superoxide radical anion, and the spontaneous decomposition of protonated ONOO^–^, respectively [32,33,34].

H_2_O_2_ + Fe^2+^ → Fe^3+^ + HO^−^ + HO^•^ (1) (1)

HOCl + O_2_^•−^ → O_2_ + Cl^−^ + HO^•^ (2) (2)

ONOO^−^ + H^+^ ⇆ ONOOH → ^•^NO_2_ + HO^•^ (3) (3)

We used epithelial embryonic cell lines (EUE) comparing the wild type (pBD650) with the same line where the XPA gene was silenced by 80% (siXPA). Recently some of us have shown that EUE-siXPA cell lines are characterized by increased steady-state ROS levels and by an altered glutathione redox state as compared to wt [35]. By using EPR spectroscopy, the ROS levels in the EUE-siXPA cell line were found significantly increased with respect to their normal counterpart in a reaction catalyzed by transition metals [36]. In these two types of cells we tested the hypothesis that different degrees of lesions could be produced under different O_2_ tensions. In particular, we compared hypoxia (1% of O_2_) to physioxia and hyperoxia (5% and 21% of O_2_ respectively), in EUE-pBD650 (wt) and EUE-siXPA (deficient) cells. The levels of the four diastereomeric 5′,8-cyclopurine lesions (5′*S-*cdG, 5′*R*-cdG, 5′*S-*cdA, and 5′*R*-dA), and the 8-oxo-Pu lesions (8-oxo-dG and 8-oxo-dA) were determined. Furthermore, in both cell lines the levels of copper (Cu) and iron (Fe) were quantified in order to indirectly establish the potential production of ROS levels in these cells, and support the hypothesis of functional connection among metal levels, hydroxyl radical generation and oxidative DNA damage accumulation in association with oxygen concentration. To monitor the cellular response of stress conditions due to hyperoxia (21% O_2_), cell membrane compartment was considered using the fatty acid-based membrane lipidomic analysis [25,37], which is informative of lipid remodeling associated with DNA damage.

## 2. Materials and Methods

### 2.1. Materials

Nuclease P1 from *Penicillium citrinum*, phosphodieasterase I and II, alkaline phosphatase from bovine intestinal mucosa, DNase I and DNase II, benzonase 99%, BHT, deferoxamine mesylate, and pentostatin were purchased from Sigma-Aldrich (Steinheim, Germany). RNase T1 was from Thermo Fisher Scientific (Waltham, MA, USA) and RNase A from Roche Diagnostic GmbH, (Mannheim*,* Germany). 2′-Deoxyadenosine monohydrate and 2′-Deoxyguanosine monohydrate were purchased from Berry & Associates Inc. (Dexter, NY, USA). Isotopic labelled internal standards of 5′*R*-cdA, 5′*S*-cdA, 5′*R*-cdG, 5′*S*-cdG, 8-oxo-dA, and 8-oxo-dG were prepared according to the previously reported procedures [38]. Solvents (HPLC-grade) were purchased from Fisher Scientific (Waltham, MA, USA). The 3 kDa cut-off filters were obtained from Millipore (Bedford, OH, USA).

### 2.2. Cell Cultures

The EUE-siXPA and EUE-pBD650 cell lines have been previously described [8]. Briefly, the EUE-siXPA cell line presents 80% silencing of the XPA gene and is hypersensitive to the killing effects of UV. Cells were cultured in Dulbecco’s modified Eagle’s medium (DMEM, Invitrogen Life Technologies S.r.l., Milan, Italy), supplemented with 10% fetal calf serum (Corning, NY, USA). Cell lines were cultured in a CO_2_ incubator (SeriesII Water Jacket 3131, Thermo Scientific, Waltham, MA, USA) at three different O_2_ concentrations (1%, 5% and 21%) for two weeks.

### 2.3. Genomic DNA Isolation

Genomic DNA was isolated using a high-salt extraction method. A nuclei lysis buffer containing 10 mM Tris-HCl, 7.5, 400 mM NaCl, 2 mM EDTA, 1% SDS (*w/v*), 200 μg/mL proteinase K, 0.1 mM deferoxamine, 0.1 mM butylated hydroxytoluene (BHT), was used to resuspend the cells (1 × 10^7^ cells in 1 mL of lysis buffer) and the lysates were incubated at 55 °C overnight. After 16 h, 360 µL of saturated NaCl solution (12 g of NaCl and water until 50 mL final volume) was added and incubated at 55 °C for 15 min, then the mixture was centrifuged at maximum speed for 30 min. The nucleic acids, in the supernatant, were precipitated with 2.5 volumes of absolute ethanol, incubated at −20 °C for 30 min and then centrifuged for 30 min at maximum speed. Nucleic acids were resuspended in a suitable volume and RNase A (20 μg/mL) was added. After 1h incubation at 37 °C, an extraction step with an equal volume of chloroform/isoamyl alcohol (24:1, *v/v*) and ethanol precipitation steps were performed. After centrifugation at maximum speed for 10 min, the resulting DNA pellet was washed twice with 70% cold ethanol, allowed to air-dry and resuspended in water.

### 2.4. Enzymatic Digestion Protocol

An aliquot of 10 μg isolated DNA was dissolved in 100 μL of Ar flushed 10 mM Tris-HCl (pH 7.9), containing 10 mM MgCl_2_, 50 mM NaCl, 0.2 mM pentostatin, 5 μM BHT, and 3 mM deferoxamine and the internal standards were added ([^15^*N*_5_]-5′*S*-cdA, [^15^*N*_5_]-5′*R*-cdA, [^15^*N*_5_]-5′*S*-cdG, [^15^*N*_5_]-5′*R*-cdG, [^15^*N*_5_]-8-oxo-dG and [^15^*N*_5_]-8-oxo-dA) as previously described (see Figure S1) [25,31]. Benzonase (3 U in 20 mM Tris-HCl pH 8.0, 2 mM MgCl_2_ and 20 mM NaCl), 4 mU phosphodiesterase I, 3 U DNAse I, 2 mU of phosphodiesterase II and 2 U of alkaline phosphatase were added and the mixture was incubated at 37 °C. After 21 h, 35 μL of Ar flushed buffer containing 0.3 M AcONa (pH 5.6) and 10 mM ZnCl_2_ were added along with 0.5 U of Nuclease P1 (in 30 mM AcONa pH 5.3, 5 mM ZnCl_2_ and 50 mM NaCl), 4 mU PDE II and 125 mU of DNAse II and the mixture was further incubated at 37 °C for extra 21 h. A step-quenching with 1% formic acid solution (final pH~7) was followed, the digestion mixture was placed in a microspin filter (3 kDa) and the enzymes were filtered off by centrifugation at 14,000× *g* (4 °C) for 20 min. Subsequently, the filtrate was freeze-dried before HPLC analysis, clean-up, and enrichment.

### 2.5. Measurement of Modified Nucleosides by LC-MS/MS

The samples were analyzed by an HPLC-UV system coupled with a sample collector, while the fractions containing the lesions were collected, freeze-dried, pooled, freeze-dried again, redissolved in Milli-Q water and subsequently injected to the LC-MS/MS system [13,38,39,40]. A triple-stage quadrupole mass spectrometer equipped with electrospray ionization (ESI) source in positive mode was employed for the detection and quantification of the lesions in the enzymatically digested DNA samples. The gradient elution program used for the chromatographic separation of the DNA lesions initiated with 99% of 2 mM ammonium formate (solvent A) and 1% acetonitrile (solvent B) (held for 1 min), increasing solvent B from 1% to 9.8% within 20 min and then immediately to 15% solvent B (held for 5 min), closing with initial conditions for 10 min re-equilibration. The flow rate remained constant at 0.2 mL/min, the injection volume was 30 μL and the column temperature was set at 30 °C. Detection was performed in multiple reaction monitoring mode (MRM) using the two most intense and characteristic precursor/product ion transitions for each DNA lesion (Figure S2 and Table S1).

### 2.6. Metals Quantification

For determination of copper (Cu) and iron (Fe) the wt and XPA-defective EUE cell pellets were subjected to a mineralization cycle in ModBlock plate (ModBlock CPI International, Santa Rosa, CA, USA) with 100 µL of HNO_3_ for 15 min at 60–70 °C and, at the end, 400 µL of ultra-pure deionized water (Barnstead EASY-PureII, Dubuque, IA, USA) were added. The quantification was performed using iCAP Q Inductively Coupled Plasma Mass Spectrometer (ICP-MS) equipped with the collision cell pressurized with He (Thermo Fisher Scientific, Bremen, Germany) in the KEDS mode. The instrument configuration and operation parameters are shown in Table S10. The iCAP Q was equipped with a PFA-ST MicroFlow nebulizer (ESI, Omaha, NB, USA), a Peltier cooled quartz spray chamber (operating at 3 °C), a 2.0 mm ID sapphire injector and a demountable quartz torch with interface Ni sampler and skimmer.

Prior to the analysis a volume of 200 µL was diluted (1:2 *v/v*) with ultra-pure deionized water and Cu and Fe quantification was carried out through calibration curve in the range 0.25–50 ng/mL for each element and using in as internal standard (IS) (1 ng/mL in the analytical solutions). The calibrants and the IS solution were daily prepared from certified solutions of 1 mg/mL (CPAchem Ltd., Stara Zagora, Bulgaria). Accuracy of the analytical procedure was assessed using the Certified Reference Material (CRM) Seronorm Lyophilized Human Serum (Sero, Billingstad, Norway) at concentration level II, resulting in the range 82–84% for the two metals. Details about validation results are reported elsewhere [36].

The limit of detection (LoD) was calculated following the criterion of the 3 sigmas, analyzing 10 samples of the blank. The resulting LoDs for Cu and Fe were 0.072 and 0.734 ng/mL, respectively.

### 2.7. Lipid Extraction and Fatty Acid-Based Lipidomic Analysis

Cell membrane phosholipids were isolated using the well-established Folch method [41]. Briefly, the pellet was re-suspended in pure water and lipids were extracted with 2:1 chloroform:methanol and examined by thin layer chromatography (n-hexane/diethyl ether/acetic acid 70/30/1) to determine the purity of the phospholipid fraction. The phospholipid extract was then treated with 0.5 M KOH/MeOH for 10 min at room temperature under stirring for the derivatization of fatty acid residues of the phospholipids into their corresponding fatty acid methyl esters (FAME). FAME were extracted with n-hexane, n-hexane phase was dehydrated with anhydrous Na_2_SO_4_, evaporated and analyzed by Agilent 7890B CG system equipped with a 60 m × 0.25 mm × 0.25 μm (50%-cyanopropyl)-methylpolysiloxane column (DB23, Agilent, Santa Clara, CA, USA), a flame ionization detector (FID), with injector temperature at 230 °C and split injection 50:1. Oven temperature started from 165 °C, held for 3 min, followed by an increase of 1 °C/min up to 195 °C, held for 40 min, followed by a second increase of 10 °C/min up to 240 °C, and held for 10 min. A constant pressure mode (29 psi) with helium as the carrier gas was used. Methyl esters were identified by comparison with the retention times of commercially available standards or trans fatty acid references, obtained as described elsewhere) as described previously [42].

### 2.8. Statistical Analysis

All measurements were performed in triplicate and the data were expressed as mean ± standard deviation (SD). The unpaired *t*-test was used for statistical analysis and a two-tailed *p*-value < 0.05 and *p*-value < 0.005 were considered to indicate a statistically significant difference.

## 3. Results

### 3.1. Levels of cPu and 8-oxo-Pu in DNA Samples

**Figure 3:**
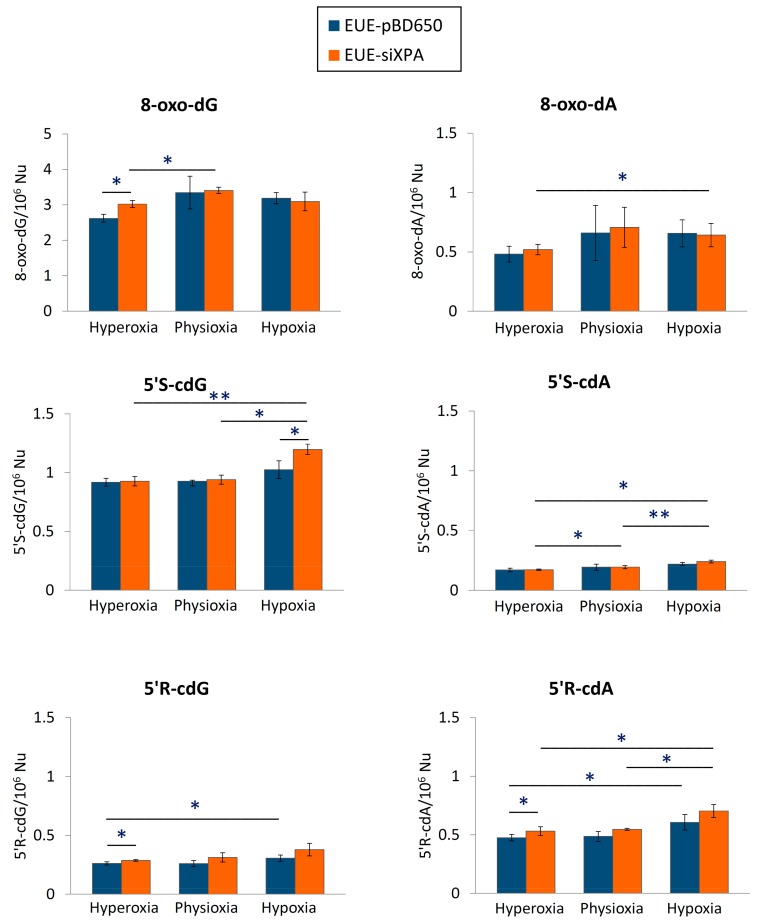
The levels (lesions/10^6^ nucleosides) of 8-oxo-dG and 8-oxo-dA, 5’*R*-cdG, 5’*S*-cdG, 5’*R*-cdA and 5’*S*-cdA in DNA samples isolated from EUE-pBD650 and EUE-siXPA (wt and deficient, respectively) cells in hyperoxic, physioxic and hypoxic conditions. Error bars: standard deviation of the mean, calculated from three independent samples, *: statistically significant difference (*p* < 0.05) between the groups, **: statistically significant difference (*p* < 0.005) between the groups.

LC-MS/MS analysis revealed that both EUE-pBD650 (wt) and EUE-siXPA (deficient) cells accumulated high levels of cPu under hypoxic conditions (1%) compared with physioxic (5%) and hyperoxic conditions (21%) (Figure 3). In Supplementary Materials Table S2, Table S3, and Table S4 collected all the experimental values and statistical analysis. Under all different oxygen concentrations, the 5′*S*-cdG was found the most predominant with detected levels of 0.92-1.03/106 nucleosides in wt cells and 0.93-1.20/106 nucleosides in deficient ones (see Tables S2 and S3). Significantly elevated levels of 5′*R*-cdG (*p* = 0.033) and 5′*R*-cdA (*p* = 0.032) were observed in EUE-pBD650 cells under hypoxia (blue bars vs. orange bars) while 5′*R*-cdA, 5′*S*-cdG and 5′*S*-cdA were found significantly increased in EUE-siXPA cells under hypoxic conditions (*p* = 0.037, *p* = 0.002, *p* = 0.020, respectively). It is worth underling that, in EUE-siXPA cells, we found statistically significant alterations comparing physioxic and hypoxic conditions in the levels of 5′*R*-cdA (*p* = 0.028), 5′*S*-cdG (*p* = 0.025) and 5′*S*-cdA (*p* = 0.003) in EUE-siXPA cells (Figure 3). Moreover, comparison of wild type and deficient cell lines, revealed statistically significant differences have been indicated in the levels of 5′*R*-cdG (*p* = 0.019) and 5′*R*-cdA (*p* = 0.022) under hyperoxic conditions (blue bars *vs* orange bars) as well as in the levels of 5′*S*-cdG (*p* = 0.013) under hypoxic conditions (blue bar *vs* orange bar, Figure 3).

Figure 3 also illustrates the levels of 8-oxo-Pu (8-oxo-dG and 8-oxo-dA) in genomic DNA isolated from EUE-pBD650 and EUE-siXPA cells in physioxic, hyperoxic and hypoxic conditions (Table S2 and S3 collect the levels of 8-oxo-Pu). The 8-oxo-dG level was found significantly elevated in deficient cells compared to the wild type cell line (*p* = 0.015) in hyperoxic conditions (blue bars vs. orange bars). Moreover, EUE-siXPA cells are characterized by a significant enhancement of this lesion comparing hyperoxic and physioxic conditions (*p* = 0.042). Furthermore, deficient cells significantly accumulated 8-oxo-dA under hypoxic conditions (*p* = 0.033).

**Figure 4:**
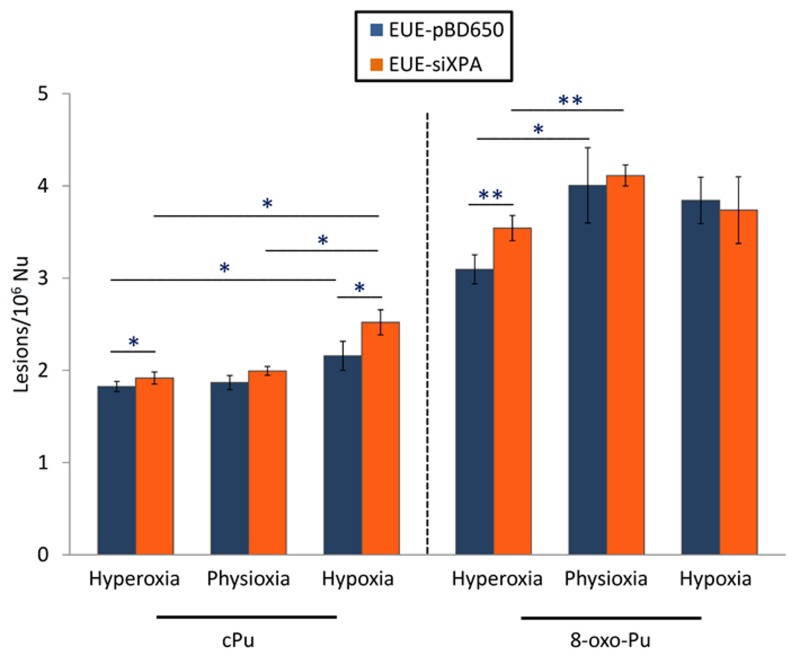
The levels (lesions/10^6^ nucleosides) of sum of cyclopurine and 8-oxo-purine lesions in DNA isolated from EUE-pBD650 (wt) and EUE-siXPA (deficient) cells in in hyperoxic, physioxic and hypoxic conditions. Error bars: standard deviation of the mean, calculated from three independent samples, *: statistically significant difference (*p* < 0.05) between the groups, **: statistically significant difference (*p* < 0.005) between the groups.

Total 8-oxo-Pu levels were determined approximately 1.5-2-fold more elevated compared with total cPu as illustrated in Figure 4 (see Table S5 for detailed values). For the first type of lesions (8-oxo-dG), statistically, significant differences were found under hyperoxic conditions among wild type and deficient cells (*p* = 0.002) as well as among hyperoxia and physioxia both in EUE-pBD650 and EUE-siXPA cells (*p* = 0.026 and 0.001, respectively. See Table S6 for detailed values). On the other side, total cPu have been observed statistically significant higher in deficient cell lines than wt cells under hyperoxic (*p* = 0.027) and hypoxic conditions (*p* = 0.009). Moreover, the levels of these lesions were identified significantly altered in EUE-pBD650 cells comparing hyperoxia and hypoxia (*p* = 0.047) and in EUE-siXPA cells among physioxia and hypoxia (*p* = 0.037), as presented in Table S6. It should be highlighted the statistical significance among total cPu and 8-oxo-Pu and oxygen concentration, statistically significant alterations have been found either in wild type cell line under hyperoxia (*p* = 0.009), physioxia (*p* = 0.016) and hypoxia (*p* = 0.001) or in deficient cell (*p* = 0.005 for hyperoxia, *p* = 0.001 for physioxia, *p* = 0.016 for hypoxia (Table S7).

In Table S8, Table S9 and Figure S3, the levels of cdG and cdA together with 8-oxo-dG and 8-oxo-dA lesions in both EUE-pBD650 and EUE-siXPA cells under the three distinct experimental conditions are presented. cdG lesions values were found approximately 1-fold higher compared with cdA levels either in wild type or in a deficient cell line. Among hyperoxia and hypoxia statistically significant alterations have been observed in deficient cells for cdG (*p* = 0.010), cdA (*p* = 0.012) and 8-oxo-dA (*p* = 0.033) as well as in wild type cells for cdA (*p* = 0.037). It should be highlighted that EUE-siXPA cells accumulated higher levels of both cyclopurines compared to EUE-pBD650 cells under hypoxic conditions (*p* = 0.009 for cdG, *p* = 0.008 for cdA. Similar trend was followed under hyperoxic conditions for cdA (*p* = 0.033) and 8-oxo-dG (*p* = 0.015) (Tables S8 and S9).

In Table 1, the ratios of 5’*R*/5’*S* for cdG and cdA in DNA isolated from wt and deficient cells under hyperoxic, physioxic and hypoxic conditions are reported as additional information on structural features associated to the two different diastereisomeric lesions, which are studied for their significance in the formation and repair of these lesions. The 5’*R*/5’*S* ratio is almost the same for cdG and cdA lesions under the three distinct experimental conditions, with a slight increase going from wt to deficient cell lines, being cdA approximately 9-folds higher than the corresponding value of cdG.

### 3.2. Iron and Copper Levels in XPA Cells

**Figure 5:**
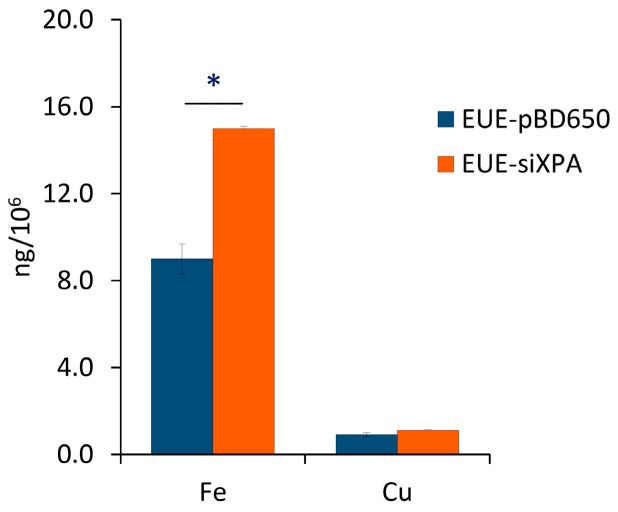
Iron and copper levels in XPA-defective cells and in their normal counterpart, quantified by ICP-MS.

As additional information connected with the role of transition metals in the radical-based biological reactivity, the levels of iron and copper in XPA-defective cells and in their normal counterpart have been quantified, following previously reported methods [43,44,45]. Analysis of experimental data indicated that Fe levels, but not Cu levels, are significantly higher (p < 0.05) in XPA-defective compared to wt cells (Figure 5).

### 3.3. Lipidomic Analysis of EUE-pBD650 and EUE-siXPA Cells Grown at 21% Oxygen

**Figure 6:**
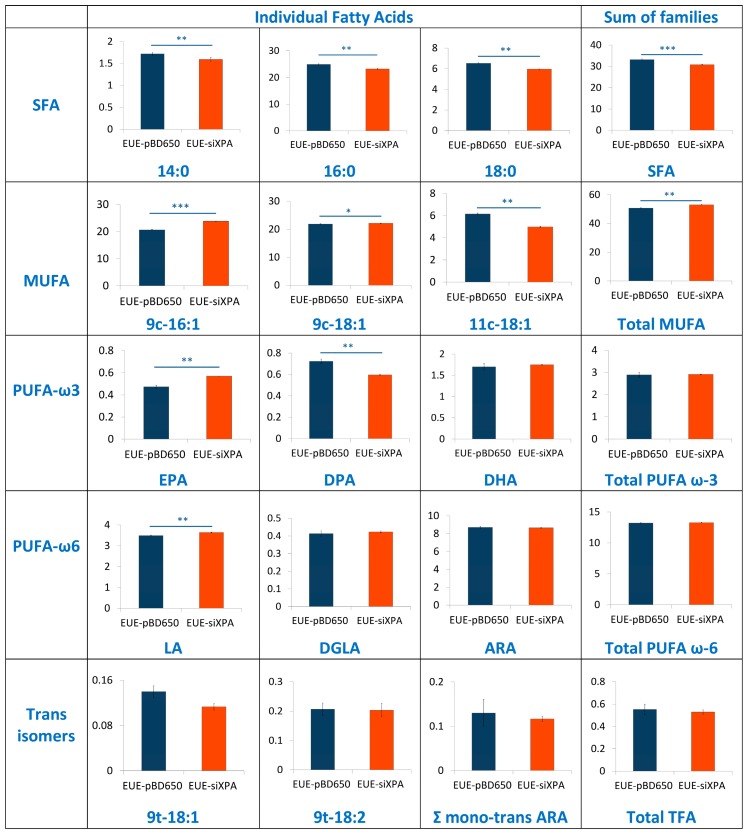
Percentage differences presenting comparison between EUE-pBD650 (wt) and EUE-siXPA cell membranes. The values are given as mean ± SD (*n* = 3). Each member of the fatty acid family is given in a row, the last column is the sum of the corresponding fatty acid family. Values significantly different from wt cells: (*) *p* < 0.05, (**) *p* < 0.01, (***) *p* < 0.001. For specific values see Table S12. Asterisks indicate the significance of differences between wt and deficient cells.

**Figure 7:**
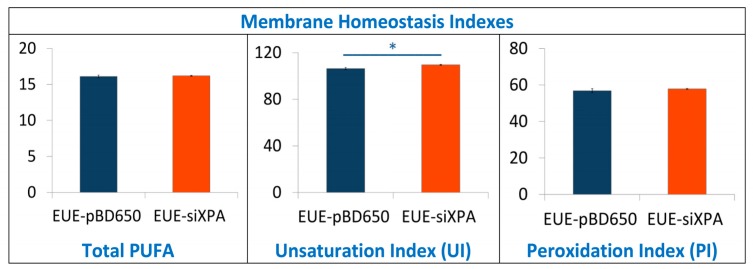
Membrane homeostasis indexes in EUE-pBD650 (wt) and EUE-siXPA cells. The values are given as mean ± SD (*n* = 3). Values significantly different from EUE-pBD650 cells: (*) *p* < 0.05, For specific values see Table S12.

To investigate the role of membrane lipids, we have analyzed membrane fatty acid composition of EUE-pBD650 and EUE-siXPA cells. Stress conditions are known to induce the fatty acid remodeling according to the Land’s cycle [46], therefore it is relevant to understand which are the differences between wt and deficient cells. Following known methodologies for the membrane pellet formation, phospholipid isolation and transesterification we obtained fatty acid methyl esters (FAME), analyzed by gas chromatography (GC). The methods used in this work for GC analysis have been described elsewhere [37,47]. Three samples for each group were analyzed. Tables S11 and S12 indicate the membrane fatty acid composition, the main membrane fatty acid families and the fatty acid indexes were calculated from these values. Fatty acid quantities are reported as relative percentages of the main peak areas obtained from the GC analyses, calibrated and recognized with appropriate references, as already described [25,48]. In Figure 6 and Figure 7 the main fatty acids and families together with the indexes are graphically represented as percentage differences, obtained by comparing wild type (EUE-pBD650) and deficient (EUE-siXPA) cells, with their statistical significance. Table S12 depicts the levels of each fatty acid type present in the membrane phospholipids of the two cell lines. For saturated fatty acids (SFA) statistically significant decrease of myristic (14:0), palmitic (16:0) and stearic (18:0) acid residues was observed comparing EUE-pBD650 to EUE-siXPA cells (*p* < 0.05, *p* < 0.01, *p* < 0.01, respectively). On the other hand, the level of monounsaturated fatty acids (MUFA) was increased in deficient cells as shown for 9c-16:1 (palmitoleic acid) and 9c-18:1 (oleic acid) (9c-16:1, *p* < 0.001, 9c-18:1, *p* < 0.05). The decrease of 11c-18:1 (vaccenic, *p* < 0.01) was observed (Figure 6). In the polyunsaturated fatty acid (PUFA) omega-3 series, EPA (5c,8c,11c,14c,17c-20:5) was found distinctly enhanced in EUE-siXPA compared with wild type cells (*p* < 0.01), whereas DPA (7c,10c,13c,16c,19c-22:5) was significantly decreased (*p* < 0.01) and DHA levels remain undisturbed. However, the total omega-3 PUFA content in membrane phospholipids did not change. No significant changes were found in the PUFA omega-6 and trans isomers. The variation found in MUFA led to the increase of the unsaturation index (UI) of EUE-siXPA cell lines compared to EUE-pBD650 cells (*p* < 0.05) (cfr., Table S12 and Figure 7).

## 4. Discussion

In this article, we analyzed three biochemical features comparing the wild type human epithelial embryonic cell line (pBD650-EUE) with its XPA-deficient counterpart (EUE-siXPA), characterized by 80% silencing of XPA and so defective in nucleotide excision repair system. Under normal oxygen incubation conditions (21% O_2_), we analyzed levels of metals (iron and copper) and membrane fatty acid balance, whereas we measured genomic DNA damage expressed as levels of cPu and 8-oxo-Pu lesions by varying oxygen concentration (21%, 5% and 1% O_2_). With the first two information we could get some insights into two important compartments contributing to cell stress response and cooperating in reactivity to and signaling of the overall damage to DNA. For what concern to metals, it is known that, in order to avoid its heavy toxicity, iron homeostasis is accurately controlled by cells, finely regulating its absorption, recycling and mobilization by specific intracellular storage. However, pathological conditions involving oxidative stress have been reported to favor the release of free iron from metal-containing molecules [32,49]. This event leads in turn to the amplification of ROS release, including the formation of strong oxidant species, including HO^•^ radical. The high reactivity and the short half-life make ^•^OH the most reactive and toxic oxidant species able to react with almost all biological molecules (proteins, lipids, carbohydrates, and nucleic acids). We found that XPA defective cells have a high Fe content compared to wt cells, which can indicate an unbalance of Fe homeostasis and higher propensity to oxidative damage than normal cells. We carried out also an evaluation of membrane lipid response to 21% O_2_ conditions, using the fatty acid monitoring of membrane phospholipids by a well-known methodology for isolation, work-up, and GC analysis. The differences in membrane lipidomic data between the wild type and XPA-defective cells pointed the attention to metabolic changes that involve the desaturase enzymatic step (stearoyl CoA-desaturase, SCD1). In fact, the increase of MUFA is due to the activation of such enzymatic pathway using the SFA pool, in particular palmitic and stearic acids which are correspondently diminished.

Desaturase enzymes use iron and oxygen as their cofactors for the double bond formation [50] and it is remarkable that we found iron levels higher in XPA-defective cells (see Figure 3). The increase of MUFA and the correspondent UI level could also produce other changes in the membrane asset and properties, increasing for example membrane fluidity. The increased fluidity with change of unsaturation is in relationship with stress signaling in cells [51] and higher ROS production is a well-known stress condition where cells can activate several responses [52]. Indeed, by the changes observed in our case, we could estimate that cell membrane is a significant “tool” to evaluate stress response. In the same conditions, the levels of all DNA lesions were significant (see Figure 4), thus underlining that different compartments must be evaluated in order to have a holistic view of the stress response. Our results also suggest that the parallel analyses of DNA-membrane compartments can be used in DNA repair enzyme-defective cells, to better understand connections with signaling cascades departing from membrane lipids, taking into account that in our case changes for the omega-3 and not for the omega-6 fatty acids were seen.

The core experiments of this paper concerned the accurate cPu lesions’ determination and quantification under different oxygen conditions. It is worth underlining that usually 21% oxygen is used as “normoxic” incubation condition, but we evaluated this condition as “hyperoxia”. As pointed out by Al-ani et al. [53], the information on the oxygen concentration in cell culture incubation is a relevant aspect to detail in the experimental methodology. In fact, our results contribute to a better understanding of the role of oxygen incubation conditions when DNA damage and repair abilities are involved. A second methodological point to remark is the individuation of the best analytical strategy including the experimental procedures for DNA extraction, hydrolysis, and derivatization. Our protocol for the quantification of cPu lesions via LC-ESI-MS/MS, in particular using isotopic labeled lesions (Figure S1), enhances the reliability of the analysis and increases to a great extent the characteristics of reproducibility and recovery of the quantification protocol [13,38]. The main findings of our study were: (i) the four cPu levels are similar in hyperoxic and physioxic conditions for both wt and deficient cell lines, whereas 8-oxo-Pu increases in all cases, (ii) both wt and deficient cell lines accumulated high levels of cPu under hypoxic compared to physioxic conditions, whereas the 8-oxo-Pu levels show an opposite trend, (iii) the diastereoisomeric ratios 5′*R*/5′*S* are independent of oxygen concentration being 0.29 for cdG and 2.69 for cdA for EUE-pBD650 (wt) and 0.32 for cdG and 2.94 for cdA for EUE-siXPA (deficient), see Table 1. Our data show for the first time the connection between oxygen concentration and DNA repair highlights of different lesions in XPA-deficient conditions and contribute to highlight the significance of cPu.

In this paper, the use of XPA defective cells allowed for targeting the ability of DNA repair and this was estimated under three oxygen concentrations, where 1% and 21% represent the hypoxia and hyperoxia conditions compared to 5% O_2_. cPu were confirmed to be a reliable marker of the oxygen tension, being their levels increased when hypoxia conditions are used. However, it is also important that when there is an NER defective condition the levels of the cPu lesions are increased thus in agreement with the fact that they are specific markers in NER defective diseases. On the other hand, 8-oxo-Pu lesions are less specific, being repaired by BER and derived in minor quantity from HO^•^ radicals and in major quantity from other oxidizing species like H_2_O_2_, peroxyl radicals, single oxygen, ONOO^–^ etc., which can be activated under hypoxia conditions. It is worth mentioning that our defective cell line was 80% silenced for repair enzymatic capability, therefore a certain amount of repair can occur.

Regarding the cPu diastereoisomers, the 5′*R*/5′*S* ratio was found to be higher in cdA than in cdG in both cell lines and at all the tested O_2_ concentrations. Moreover, starting from the knowledge that 5′*S*-cdG is a better NER substrate than 5′*S*-cdA lesion [14,15,16], this can explain the significant differences found in our experiments between these two markers in XPA-silenced cells (Figure 3). Working with HeLa cells, it was observed that 5′*R*-cdA and 5′*R*-cdG are better NER substrates that the 5′*S*-ones, while molecular dynamic simulations studies confirmed that the *R*-stereoisomers are significantly more dynamic and more distorting that the *S*-stereoisomeric lesions [14,15,16]. This result in the diastereomeric accumulation is different from the HeLa cells and breast cancer cells indicating that the DNA repair systems can have different specificity depending on tissue types. We recall that quantification of the four cPu lesions has been previously determined in estrogen receptor-alpha positive (ER-a) MCF-7 and triple negative MDA-MB-231 breast cancer cell lines before and after exposure to two different conditions: ionizing radiations and hydrogen peroxide, followed by an interval period to allow DNA repair [39]. The higher levels accumulation of 5′*S*-cdG in MDA-MB-231 and MCF-7 cells were in accordance with NER efficiencies [39].

## 5. Conclusions

In this work we show for the first time the connection of oxygen concentration in DNA repair-deficient cells, highlighting the quantity and quality of purine lesions. In particular, we checked the levels of cPu, specific markers of HO^•^ radical damage, and the 8-oxo-Pu which can be derived from other oxidizing species. The presence of a high Fe content in XPA defective cells (EUE-siXPA) can be an important indicator of the propensity to give an intense cellular stress response. Lipidomic monitoring of cell membrane under hyperoxic conditions and the resultant enhancement of MUFA together with the correspondent UI levels indicate for the first time that specific lipidome membrane alteration occurs under XPA-deficient conditions. Our results reveal a combined scenario between membrane lipid remodeling and oxidatively-induced DNA adducts under variable radical reactivity and stress status.

## Figures and Tables

**Table 1:** 5’*R*/5’*S* ratio of cPu lesions in DNA isolated from EUE-pBD and EUE-siXPA cells in hyperoxic, physioxic and hypoxic conditions.

Samples	Conditions	cdG 5’R/5’S	cdA 5’R/5’S
EUE-pBD650	Hyperoxic	0.29 ± 0.01	2.80 ± 0.38
EUE-pBD650	Physioxic	0.28 ± 0.03	2.51 ± 0.18
EUE-pBD650	Hypoxic	0.30 ± 0.01	2.77 ± 0.35
EUE-siXPA	Hyperoxic	0.31 ± 0.01	3.09 ± 0.30
EUE-siXPA	Physioxic	0.33 ± 0.04	2.81 ± 0.19
EUE-siXPA	Hypoxic	0.32 ± 0.03	2.92 ± 0.29

## Supplementary Materials

The following are available online at https://www.mdpi.com/2073-4409/8/11/1377/s1, Figure S1: ^15^N_5_ isotopically labeled of cPu and 8-oxo-Pu, Figure S2: Calibration curves for the quantification of the lesions, Figure S3: The levels of cPu and 8-oxo-Pu lesions in DNA isolated from EUE-pBD650 (wt) and EUE-siXPA (deficient) cells in hyperoxic, physioxic and hypoxic conditions, Table S1: A list of MRM transitions employed for the quantifications of DNA lesions and their corresponding stable isotope-labeled standards, Table S2 and Table S3: The levels of cPu and 8-oxo-Pu in DNA samples isolated from EUE-pBD650 (wt) and EUE-siXPA (deficient) cells in hyperoxic, physioxic and hypoxic conditions, Table S4: Values of t-Test by comparing the means of lesions in each condition, Table S5: Total amount of cPu and 8-oxo-Pu lesions in DNA isolated from EUE-pBD650 and EUE-siXPA cells in hyperoxic, physioxic and hypoxic conditions, Table S6: Values of *t*-Test by comparing the means of lesions in each condition, Table S7: Values of *t*-Test by comparing the sum of cPu and 8-oxo-Pu in each condition, Table S8: Total amount of cPus and 8-oxo-purine lesions in DNA isolated from EUE-pBD650 and EUE-siXPA cells in in hyperoxic, physioxic and hypoxic conditions, Table S9: Values of *t*-Test by comparing the means of lesions in each condition. Table S10: Instrument configuration and operation parameters of iCAP Q Inductively Coupled Plasma Mass Spectrometer (ICP-MS) equipped, Table S11: Fatty acids obtained from EUE-pBD650 (wt) and EUE-siXPA cell membrane phospholipids, Table S12: Relative percentage of fatty acid methyl esters and families from EUE-pBD650 (wt) and EUE-siXPA.
