# Supplementary material for: Purine DNA Lesions at Different Oxygen Concentration in DNA Repair-Impaired Human Cells (EUE-siXPA)
Source: Cells. 2019 Nov 1;8(11):1377. doi: 10.3390/cells8111377 (PMC6912421; doi:10.3390/cells8111377)
Supplement: Supplementary file 1 [file cells-08-01377-s001.pdf]

cells

Supplementary Material

Purine DNA lesions at different oxygen concentration in DNA repair-impaired human cells (EUE-siXPA)

Marios G. Krokidis, Eleonora Parlanti, Maria Rosaria D'Errico, Barbara Pascucci, Anna Pino, Alessandro Alimonti, Donatella Pietraforte, Annalisa Masi, Carla Ferreri and Chryssostomos Chatgilialoglu*

* Correspondence: chrys@isof.cnr.it; Tel.: +39-051-639-8309

Table of Contents

Figure S1	page 2
Figure S2	page 3
Table S1	page 4
Table S2 and Table S3	Page 5
Table S4 and Table S5	Page 6
Table S6, Table S7 and Table S8	Page 7
Table S9 and Figure S3	Page 8
Table S10 and Table S11	Page 9
Table S12	Page 10

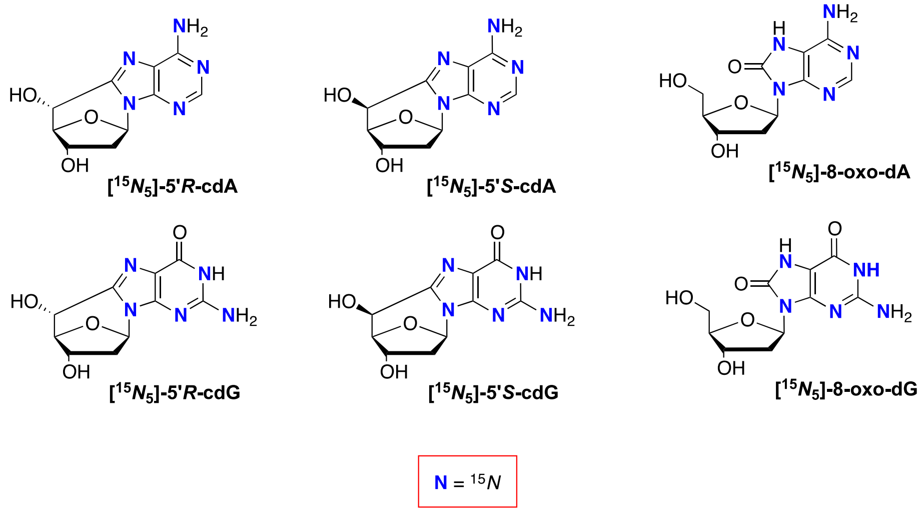

Figure S1. $^{15}N_5$ isotopically labeled of 5′,8-cyclo-2′-deoxyadenosine (cdA) and 5′,8-cyclo-2′-deoxyguanosine (cdG) in their 5'*R* and 5'*S* diastereomeric forms, as well as 8-oxo-2′-deoxyadenosine (8-oxo-dA) and 8-oxo-2′-deoxyguanosine (8-oxo-dG)

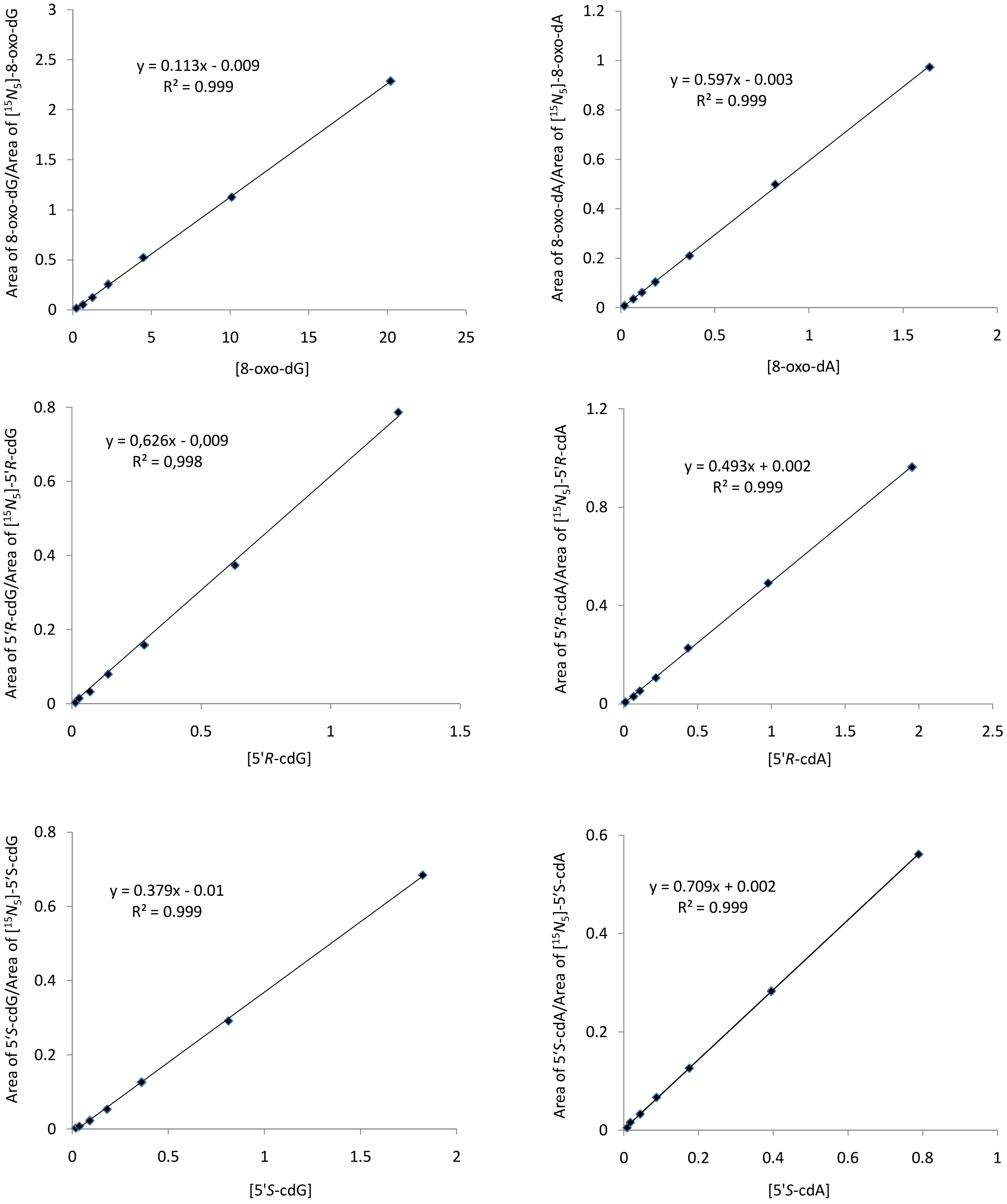

Figure S2. Calibration curves for the quantification of the lesions (nM)

Table S1. A list of MRM transitions employed for the quantifications of the six oxidatively induced DNA lesions and their corresponding stable isotope-labeled standards.

Lesions	Precursor ion m/z	Product ion m/z	Collision energy (V)
5'*R*-cdA	250	164	14
[^{15}*N*$_5$]-5'*R*-cdA	255	169	14
5'*S*-cdA	250	164	16
[^{15}*N*$_5$]- 5'*S*-cdA	255	169	16
5'*R*-cdG	266	180	18
[^{15}*N*$_5$]- 5'*R*-cdG	271	185	18
5'*S*-cdG	266	180	16
[^{15}*N*$_5$]- 5'*S*-cdG	271	185	16
8-oxo-dA	268	152	19
[^{15}*N*$_5$]- 8-oxo-dA	273	157	19
8-oxo-dG	284	168	18
[^{15}*N*$_5$]- 8-oxo-dG	289	173	18

Table S2: The levels (lesions/10^6 nucleosides) of 5'*R*-cdG, 5'*S*-cdG, 5'*R*-cdA, 5'*S*-cdA, 8-oxo-dG and 8-oxo-dA in DNA samples isolated from EUE-pBD650 (wt) and EUE-siXPA (deficient) cells in hyperoxic, physioxic and hypoxic conditions. The numbers represent the values of DNA lesions levels from the measurement of each sample.

Sample	Condition	5'*R*-cdG	5'*R*-cdA	5'*S*-cdG	5'*S*-cdA	8-oxo-dG	8-oxo-dA
EUE-pBD650	hyperoxia	0.256	0.495	0.936	0.168	2.504	0.500
EUE-pBD650	hyperoxia	0.277	0.486	0.938	0.156	2.596	0.408
EUE-pBD650	hyperoxia	0.253	0.444	0.880	0.186	2.740	0.537
EUE-pBD650	physioxia	0.274	0.535	0.921	0.221	2.832	0.845
EUE-pBD650	physioxia	0.279	0.460	0.920	0.191	3.477	0.400
EUE-pBD650	physioxia	0.230	0.464	0.937	0.170	3.727	0.736
EUE-pBD650	hypoxia	0.293	0.632	0.967	0.204	3.181	0.561
EUE-pBD650	hypoxia	0.338	0.656	1.110	0.232	3.346	0.783
EUE-pBD650	hypoxia	0.289	0.532	0.997	0.221	3.033	0.624
EUE-siXPA	hyperoxia	0.285	0.568	0.905	0.166	3.008	0.485
EUE-siXPA	hyperoxia	0.295	0.533	0.973	0.177	2.930	0.506
EUE-siXPA	hyperoxia	0.281	0.493	0.902	0.173	3.128	0.570
EUE-siXPA	physioxia	0.356	0.541	0.938	0.179	3.508	0.554
EUE-siXPA	physioxia	0.281	0.557	0.903	0.200	3.355	0.677
EUE-siXPA	physioxia	0.302	0.541	0.981	0.204	3.355	0.890
EUE-siXPA	hypoxia	0.323	0.684	1.156	0.254	3.086	0.644
EUE-siXPA	hypoxia	0.429	0.766	1.244	0.236	3.363	0.739
EUE-siXPA	hypoxia	0.384	0.660	1.194	0.232	2.837	0.543

Table S3. The levels (lesions/10^6 nucleosides) of 5'*R*-cdG, 5'*S*-cdG, 5'*R*-cdA, 5'*S*-cdA, 8-oxo-dG and 8-oxo-dA in DNA samples isolated from EUE-pBD650 (wt) and EUE-siXPA (deficient) cells in hyperoxic, physioxic and hypoxic conditions (mean values ± standard deviation of three sample measurements).

Sample	Condition	5'*R*-cdG	5'*R*-cdA	5'*S*-cdG	5'*S*-cdA	8-oxo-dG	8-oxo-dA
EUE-pBD650	Hyperox.	0.262±0.013	0.475±0.027	0.918±0.033	0.170±0.015	2.613±0.119	0.482±0.066
EUE-pBD650	Physiox.	0.261±0.027	0.486±0.043	0.926±0.010	0.194±0.026	3.345±0.462	0.660±0.232
EUE-pBD650	Hypox.	0.307±0.027	0.607±0.066	1.025±0.076	0.219±0.014	3.187±0.157	0.656±0.115
EUE-siXPA	Hyperox.	0.287±0.007	0.532±0.040	0.927±0.040	0.172±0.006	3.022±0.100	0.520±0.044
EUE-siXPA	Physiox.	0.313±0.039	0.546±0.039	0.940±0.039	0.195±0.013	3.406±0.088	0.707±0.170
EUE-siXPA	Hypox.	0.379±0.053	0.704±0.056	1.198±0.044	0.241±0.012	3.095±0.263	0.642±0.098

Table S4. Values of t-Test (Two-Sample Assuming Unequal Variances) by comparing the means of lesions in each condition.

Samples	Conditions	5'*R*-cdG	5'*R*-cdA	5'*S*-cdG	5'*S*-cdA	8-oxo-dG	8-oxo-dA
EUE-pBD650 vs EUE-siXPA	Hyperoxia	0.019*	0.022*	0.710	0.842	0.015*	0.360
EUE-pBD650 vs EUE-siXPA	Physioxia	0.180	0.163	0.495	0.986	0.865	0.812
EUE-pBD650 vs EUE-siXPA	Hypoxia	0.077	0.052	0.013*	0.271	0.275	0.800
EUE-pBD650	Hyperox. vs Physiox.	0.965	0.626	0.771	0.362	0.070	0.224
EUE-siXPA	Hyperox. vs Physiox.	0.400	0.572	0.783	0.049*	0.042*	0.125
EUE-pBD650	Hyperox. vs Hypox.	0.033*	0.032*	0.123	0.066	0.056	0.226
EUE-siXPA	Hyperox. vs Hypox.	0.084	0.037*	0.002**	0.020*	0.759	0.033*
EUE-pBD650	Physiox. vs Hypox.	0.074	0.090	0.165	0.358	0.652	0.986
EUE-siXPA	Physiox. vs Hypox.	0.342	0.028*	0.025*	0.003**	0.195	0.690

Statistically significant samples: * <0.05 , **<0.005

Table S5. Total amount of cPu and 8-oxo-Pu lesions in DNA isolated from EUE-pBD650 and EUE-siXPA cells in hyperoxic, physioxic and hypoxic conditions.

Samples	Condition	cPu	8-oxo-Pu
EUE-pBD650	Hyperoxia	1.825±0.054	3.095±0.158
EUE-pBD650	Physioxia	1.868±0.077	4.005±0.408
EUE-pBD650	Hypoxia	2.157±0.158	3.843±0.252
EUE-siXPA	Hyperoxia	1.917±0.065	3.542±0.137
EUE-siXPA	Physioxia	1.994±0.048	4.113±0.116
EUE-siXPA	Hypoxia	2.521±0.136	3.737±0.361

Table S6. Values of *t*-Test (Two-Sample Assuming Unequal Variances) by comparing the means of lesions in each condition

Samples	Conditions	cPu	8-oxo-Pu
EUE-pBD650 vs EUE-siXPA	Hyperoxia	0.027*	0.002**
EUE-pBD650 vs EUE-siXPA	Physioxia	0.130	0.602
EUE-pBD650 vs EUE-siXPA	Hypoxia	0.009*	0.343
EUE-pBD650	Hyperoxia vs Physioxia	0.281	0.026*
EUE-pBD650	Hyperoxia vs Hypoxia	0.047*	0.074
EUE-pBD650	Physioxia vs Hypoxia	0.104	0.668
EUE-siXPA	Hyperoxia vs Physioxia	0.345	0.001**
EUE-siXPA	Hyperoxia vs Hypoxia	0.010*	0.564
EUE-siXPA	Physioxia vs Hypoxia	0.037*	0.300

Statistically significant samples: * <0.05 , **<0.005

Table S7. Values of *t*-Test (Two-Sample Assuming Unequal Variances) by comparing the sum of cPu and 8-oxo-Pu in each condition

Samples	Condition	cPu vs 8-oxo-Pu
EUE-pBD650	Hyperoxia	0.009
EUE-pBD650	Physioxia	0.016
EUE-pBD650	Hypoxia	0.001
EUE-siXPA	Hyperoxia	0.005
EUE-siXPA	Physioxia	0.001
EUE-siXPA	Hypoxia	0.016

Table S8. Total amount of cPu and 8-oxo-Pu lesions in DNA isolated from EUE-pBD650 and EUE-siXPA cells in in hyperoxic, physioxic and hypoxic conditions.

Samples	Conditions	cdG	cdA	8-oxo-dG	8-oxo-dA
EUE-pBD650	Hyperoxia	1.180± 0.043	0.645± 0.017	2.613± 0.119	0.482± 0.066
EUE-pBD650	Physioxia	1.187± 0.017	0.680± 0.067	3.345± 0.462	0.660± 0.232
EUE-pBD650	Hypoxia	1.331± 0.102	0.826± 0.068	3.187± 0.157	0.656± 0.115
EUE-siXPA	Hyperoxia	1.214± 0.047	0.704± 0.034	3.022± 0.100	0.520± 0.044
EUE-siXPA	Physioxia	1.254± 0.061	0.741± 0.018	3.406± 0.088	0.707± 0.170
EUE-siXPA	Hypoxia	1.577± 0.097	0.944± 0.055	3.095± 0.263	0.642± 0.098

Table S9. Values of *t*-Test (Two-Sample Assuming Unequal Variances) by comparing the means of lesions in each condition.

Samples	Conditions	cdG	cdA	8-oxo-dG	8-oxo-dA
EUE-pBD650	Hyperoxia vs Physiox.	0.652	0.349	0.070	0.224
EUE-pBD650	Hyperoxia vs Hypoxia	0.087	0.037*	0.056	0.226
EUE-pBD650	Physioxia vs Hypoxia	0.118	0.091	0.652	0.986
EUE-siXPA	Hyperoxia vs Physioxia	0.585	0.305	0.042*	0.125
EUE-siXPA	Hyperoxia vs Hypoxia	0.010*	0.012*	0.759	0.033*
EUE-siXPA	Physiox.vs Hypox.	0.068	0.020*	0.195	0.690
EUE-pBD650 vs EUE-siXPA	Hyperoxia	0.201	0.033*	0.015*	0.360
EUE-pBD650 vs EUE-siXPA	Physioxia	0.250	0.337	0.865	0.812
EUE-pBD650 vs EUE-siXPA	Hypoxia	0.009*	0.008*	0.275	0.800

Statistically significant samples: * <0.05

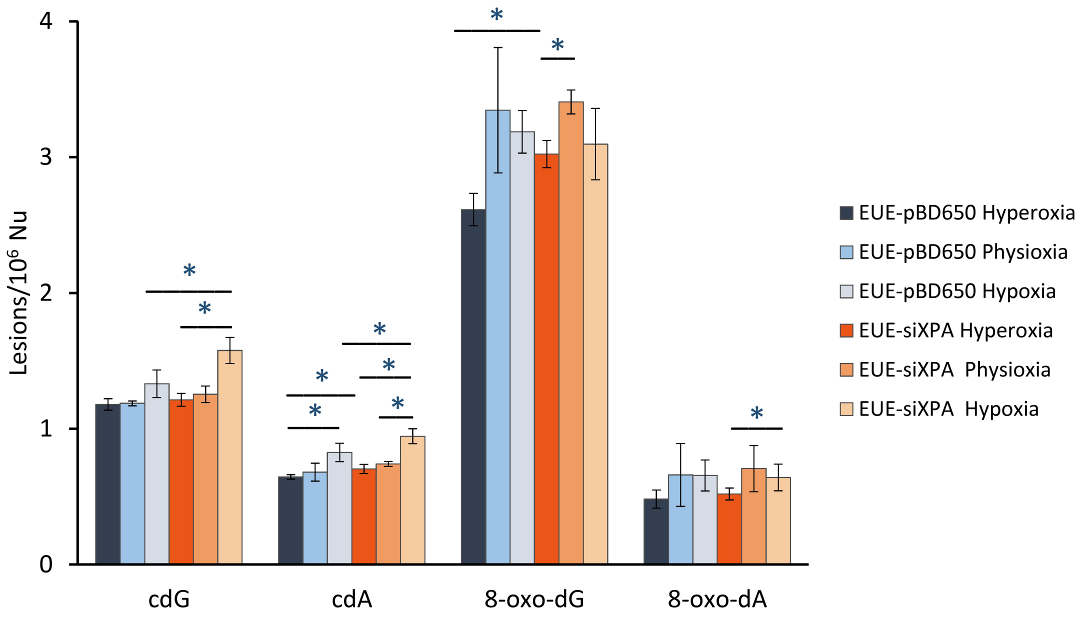

Figure S3. The levels (lesions/10^6 nucleosides) of cyclopurine and 8-oxo-purine lesions in DNA isolated from EUE-pBD650 (wt) and EUE-siXPA (deficient) cells in hyperoxic, physioxic and hypoxic conditions.

Table S10. Instrument configuration and operation parameters of iCAP Q Inductively Coupled Plasma Mass Spectrometer (ICP-MS) equipped.

RF power:	1550 w
Argon gas flow (L/min):	Plasma 14, Auxiliary 0.8, Nebulizer 1.08
Collision cell gas:	He at 1 mL/min
KED barrier:	2 V
Analytical parameters:	Dwell time 0.1 s per peak, 10 sweeps, 3 replicates
Analytical masses:	^{65}Cu, ^{56}Fe
Internal standard:	^{115}In

Table S11. Fatty acid methyl esters (FAME) obtained from each sample of EUE-pBD650 (wt) and EUE-siXPA cell membrane phospholipids. The values are reported as relative percentage (% rel) of the total FAME peak areas detected in the GC analysis, corresponding to > 98% of the total peaks present in the chromatogram.

FAME	EUE-pBD650	EUE-pBD650	EUE-pBD650	EUE-siXPA	EUE-siXPA	EUE-siXPA
14:0	1.75	1.68	1.72	1.62	1.54	1.62
16:0	25.02	24.6	25.12	23.31	23.02	23.36
16:1 trans	0.13	0.1	0	0.09	0.11	0.09
16:1-c6	1.48	1.52	1.49	1.56	1.57	1.56
16:1-c9	20.76	20.63	20.59	23.88	23.83	23.88
18:0.	6.48	6.53	6.58	5.96	5.98	5.95
18:1 trans	0.13	0.14	0.15	0.12	0.11	0.11
18:1-c9	21.81	22.03	21.77	22.08	22.24	22.03
18:1-c11	6.24	6.16	6.06	4.94	5.05	4.96
18:2-trans	0.2	0.23	0.19	0.19	0.19	0.23
18:2-w6	3.51	3.49	3.47	3.64	3.66	3.62
20:1-c11	0.32	0.33	0.35	0.28	0.28	0.27
20:2-w6	0.28	0.27	0.25	0.26	0.22	0.24
20:3-w6	0.41	0.43	0.4	0.42	0.43	0.42
20:4-w6	8.59	8.79	8.74	8.63	8.71	8.63
trans 20:4-w6	0.13	0.1	0.16	0.11	0.12	0.12
20:5-w3	0.46	0.48	0.48	0.57	0.57	0.57
22:5-w3	0.7	0.73	0.74	0.59	0.6	0.6
22:6-w3	1.61	1.74	1.75	1.75	1.76	1.74
SFA	33.25	32.81	33.42	30.89	30.54	30.92
MUFA	50.87	50.91	50.41	52.95	53.19	52.91
PUFA	15.88	16.27	16.17	16.16	16.26	16.17
PUFA w6	13.12	13.31	13.2	13.25	13.33	13.26
PUFA w3	2.77	2.95	2.97	2.91	2.93	2.91
trans	0.59	0.57	0.5	0.51	0.53	0.55
UI	105.44	107.18	106.41	109.36	110.05	109.22
PI	55.60	57.58	57.37	57.74	58.19	57.64

Table S12. Mean values (± SD) and significance (p value) of the relative percentages (% rel) of fatty acid methyl esters (FAME) and families from EUE-pBD650 (wt) and EUE-siXPA cell lines. The values are obtained from the results reported in Table S11.

FAME	EUE-pBD650	EUE-siXPA	
	n = 3[a]	n = 3[a]	*p* value[b]
14:0.	1.72 ± 0.04	1.59 ± 0.05	0.009
16:0.	24.91 ± 0.28	23.23 ± 0.18	0.001
16:1 trans	0.08 ± 0.07	0.10 ± 0.01	0.650
16:1-c6	1.50 ± 0.02	1.56 ± 0.01	0.017
16:1-c9	20.66 ± 0.09	23.86 ± 0.03	0.000
18:0.	6.53 ± 0.05	5.96 ± 0.02	0.003
18:1 trans	0.14 ± 0.01	0.11 ± 0.01	0.094
18:1-c9	21.87 ± 0.14	22.12 ± 0.11	0.006
18:1-c11	6.15 ± 0.09	4.98 ± 0.06	0.003
18:2-trans	0.21 ± 0.02	0.20 ± 0.02	0.899
18:2-w6 (LA)	3.49 ± 0.02	3.64 ± 0.02	0.006
20:1-c11	0.33 ± 0.02	0.28 ± 0.01	0.042
20:2-w6	0.27 ± 0.02	0.24 ± 0.02	0.157
20:3-w6 (DGLA)	0.41 ± 0.02	0.42 ± 0.01	0.225
20:4-w6 (ARA)	8.71 ± 0.10	8.66 ± 0.05	0.389
trans 20:4-w6	0.13 ± 0.03	0.12 ± 0.01	0.529
20:5-w3 (EPA)	0.47 ± 0.01	0.57 ± 0.00	0.005
22:5-w3 (DPA)	0.72 ± 0.02	0.60 ± 0.01	0.005
22:6-w3 (DHA)	1.70 ± 0.08	1.75 ± 0.01	0.389
SFA	33.16 ± 0.31	30.78 ± 0.21	0.001
MUFA	50.73 ± 0.28	53.02 ± 0.15	0.003
PUFA	16.11 ± 0.20	16.20 ± 0.06	0.444
PUFA w6	13.21 ± 0.10	13.28 ± 0.04	0.161
PUFA w3	2.90 ± 0.11	2.92 ± 0.01	0.775
Total Trans FA (TFA)	0.55 ± 0.05	0.53 ± 0.02	0.606
Unsaturation Index (UI)	106.34 ± 0.87	109.54 ± 0.44	0.012
Peroxidation Index (PI)	56.85 ± 1.09	57.86 ± 0.29	0.222

[a] The values are given as mean ± SD and n is the number of samples per group. [b] Significance of differences between EUE-pBD650 and EUE-siXPA cells. The unpaired t-test was used for statistical analysis and a two-tailed p-value<0.05 and p-value<0.005 were considered to indicate a statistical significant difference.
